# Secondhand smoke exposure at home and school absenteeism among children in the United States: national estimates of attributable missed school days

**DOI:** 10.1016/j.pmedr.2026.103636

**Published:** 2026-09-13

**Authors:** Yingning Wang, Hai-Yen Sung, Wendy Max, Bonnie Halpern-Felsher, Dian Gu, Sarah Planche, Chunxia Wang

**Affiliations:** a Institute for Health & Aging, School of Nursing, University of California, San Francisco, CA, United States; b Tobacco Use Prevention Education Office, California Department of Education, Sacramento, CA, United States; c Stanford REACH Lab, Division of Adolescent Medicine, Department of Pediatrics, School of Medicine, Stanford University, Palo Alto, CA, United States

**Keywords:** Secondhand smoke exposure, School absenteeism, Child health, Missed school days, Population attributable burden, National Survey of children's health

## Abstract

**Objective:**

The number of missed school days attributable to secondhand smoke (SHS) exposure at home among U.S. children has not been quantified. We estimated the association between SHS exposure at home and school absenteeism and derived national estimates of SHS-attributable missed school days.

**Methods:**

We analyzed data from the 2016–2024 National Survey of Children's Health for U.S. children aged 6–11 years. Propensity score weighting and two-part models were used to estimate the association between SHS exposure at home and school absenteeism. National estimates of SHS-attributable missed school days were derived by combining model-based attributable fractions with national public school enrollment data.

**Results:**

SHS exposure at home was associated with both a higher likelihood of missing school and a greater number of missed school days among children with any absence. In 2024, an estimated 266,008 (95% CI: 139,627, 392,390) missed school days were attributable to SHS exposure at home nationwide, corresponding to 0.95 additional missed school days per exposed child (95% CI: 0.56, 1.34).

**Conclusions:**

This study provides the first national estimates of SHS-attributable missed school days among U.S. children. These findings suggest that reducing children's SHS exposure may further improve school attendance and promote more efficient use of educational resources.

## Highlights

• Secondhand Smoke Exposure at home was associated with greater school absenteeism.
• Exposed children missed more school days than unexposed children.
• This study provides the first national estimates of attributable missed school days.
• An estimated 266,008 school days were attributable to secondhand smoke in 2024.
• Estimates used representative 2016–2024 U.S. survey data and 2024 enrollment data.

## 1 Introduction

Secondhand smoke (SHS) exposure increases children's risk of respiratory infections, asthma exacerbations, impaired lung function, and other adverse health outcomes (U.S. Department of Health and Human Services, 2014). These health effects may interfere with children's ability to attend school, making school absenteeism an important consequence of SHS exposure.

School absenteeism reflects both child health and educational disruption (Allen et al., 2018; Goldman et al., 2025). Missed school days are associated with poorer academic performance and may increase demands on educational support systems (Raghuveer et al., 2016; Daily et al., 2020). As a result, estimating the number of school days associated with preventable health risks such as SHS exposure can help quantify their broader impact beyond traditional health outcomes.

Several studies have demonstrated an association between SHS exposure at home and school absenteeism among children (Gilliland et al., 2003; Levy et al., 2011). For example, Gilliland and colleagues found higher absenteeism among SHS-exposed California children using 1996 regional data (Gilliland et al., 2003). Levy and colleagues used the 2005 National Health Interview Survey data to estimate differences in missed school days between exposed and unexposed children and the related caregiver productivity losses (Levy et al., 2011). However, these studies used older data and primarily quantified associations between SHS exposure and school absenteeism rather than estimating the number of missed school days attributable to SHS exposure at the population level. To our knowledge, no study has derived national estimates of SHS-attributable missed school days using contemporary U.S. data.

To address this gap, we examined the association between SHS exposure at home and school absenteeism for US children aged 6–11 and estimated national SHS-attributable missed school days. These estimates provide a quantitative measure of an educational consequence of SHS exposure at home and may help inform tobacco control and child health policies. Because school attendance can also affect school financing in states where funding is linked to average daily attendance (ADA), we conducted an exploratory application to California, estimating SHS-attributable missed school days and the corresponding attendance-based funding loss in California.

## 2 Methods

### 2.1 Study design and population

We used data from the 2016–2024 National Survey of Children's Health (NSCH) (U.S. Census Bureau, 2016–2024). The NSCH uses an address-based, stratified sampling design conducted separately within each state and the District of Columbia. A household screener identifies children aged 0–17 years residing in the household, after which one child is selected for an age-specific topical questionnaire completed by a parent or caregiver. When multiple eligible children are present, the probability of selection varies according to the number and characteristics of children in the household, including age and special health care needs status. The NSCH selected-child topical survey weights incorporate address-selection and within-household child-selection probabilities, as well as adjustments for nonresponse and calibration to population benchmarks. We also used the 2024 public school enrollment data from the National Center for Education Statistics at the Department of Education to derive the total number of missed school days by U.S. children aged 6–11 in 2024 (National Center for Education Statistics, 2024).

The present analysis was restricted to school-enrolled children aged 6–11 years. Most younger children are not yet enrolled in school, whereas older youth may have initiated tobacco use themselves, which is not measured in the NSCH and therefore could not be controlled for. The 2016–2024 NSCH includes 113,188 children aged 6–11 years. After excluding 1923 children with missing values for missed school days or not enrolled in school and 2235 with missing information on SHS exposure or highest adult education, the final analytic sample is 109,030 children.

### 2.2 Measures

The outcome of interest was the number of school days missed due to illness or injury in the past 12 months. Responses were originally reported in categories: 0, 1–3, 4–6, 7–10, or 11 or more days. For the primary analysis, we assigned values of 0, 2, 5, 8.50, and 11 days, respectively.

SHS exposure at home was defined based on responses to two questions. First, parents were asked whether their child lived with any household members who smoked combustible tobacco products (i.e., cigarettes, cigars, or pipe tobacco). If they answered yes, a follow-up question asked whether anyone smoked inside the home. Children were considered exposed to SHS if parents answered yes to both questions.

Based on the literature (Yao et al., 2016), covariates included child sex, age, race/ethnicity (Hispanic, non-Hispanic (NH) White, NH Black, NH Asians, and NH Other (including Multiracial)), highest education level among reported adults (less than high school; high school (including vocational, trade, or business school); some college or an associate degree; and college degree or higher), and family poverty level. Models of school absenteeism also included state and survey-year fixed effects to account for time-invariant state characteristics and shocks common across states. Race and ethnicity were based on parent- or caregiver-reported NSCH categories and treated as social, not biological, classifications. NSCH provides six completed family poverty ratio variables, known as implicates. For children with missing family-income information, these variables contain six plausible imputed poverty-ratio values. We categorized the poverty ratio within each implicate as <100%, 100%–199%, 200%–399%, or ≥ 400% of the federal poverty level (FPL). Analyses were conducted separately within each implicate, and implicate-specific estimates and variances were combined using Rubin's rules (U.S. Census Bureau, 2024). Additional details are provided in the Appendix under “ NSCH Sampling, Survey Weights, and Multiple Imputation.”

### 2.3 Statistical analysis

We described the study population and prevalence of SHS exposure using NSCH selected-child survey weights and assessed bivariate associations using Rao–Scott chi-square tests. For family poverty level, weighted estimates were calculated separately within each of the six implicates and combined using Rubin's rules; category-specific unweighted sample sizes in Table 1 represent mean counts across the six implicates.

**Table 1:** Sample distribution for children aged 6–11 in the US by missing school status in the past 12 months, National Survey of Children's Health 2016–2024.

		children aged 6–11		SHS exposure		Rao–Scott Chi-square test
		n	col%	n	row%	*P* values
All children aged 6–11		109,030	100.00	1519	1.63	
Missed school in the past 12 months	No	28,163	30.81	357	1.48	0.18
	Yes	80,867	69.19	1162	1.70	
Child sex	Female	52,537	48.90	675	1.55	0.25
	Male	56,493	51.10	844	1.71	
Child race/ethnicity	Hispanics	14,684	26.37	130	0.73	<0.01
	NH Whites	72,490	49.70	939	1.69	
	NH Blacks	6833	12.55	257	3.55	
	NH Asians	6207	4.84	31	0.39	
	NH Others	8816	6.53	162	2.13	
Parent's highest education	<HS	2614	8.72	114	2.63	<0.01
	HS	13,727	18.96	585	3.70	
	Some college or an associate degree	23,895	20.69	588	2.47	
	College or above	68,794	51.63	232	0.37	
Poverty level	0–99% FPL,	13,248	16.34	585	4.23	<0.01
	100–199% FPL,	17,952	22.85	469	2.58	
	200–399%, FPL	33,004	32.30	351	0.87	
	≥400% FPL	44,826	28.51	114	0.25	
Children's age	6	17,341	15.98	178	1.16	<0.01
	7	17,192	16.35	205	1.45	
	8	17,891	16.67	250	1.49	
	9	18,299	16.80	260	1.69	
	10	18,873	17.18	299	1.76	
	11	19,434	17.02	327	2.20	
year	2016	14,248	11.10	258	2.59	<0.01
	2017	6328	11.31	82	1.32	
	2018	9109	11.31	155	1.92	
	2019	8759	11.24	157	1.64	
	2020	12,588	10.96	193	1.76	
	2021	13,437	11.02	180	1.65	
	2022	14,753	11.06	200	1.46	
	2023	14,769	11.05	149	1.18	
	2024	15,039	10.97	145	1.16	

Note n = unweighted sample size, and % = weighted percentage based on the sampling weights alone. For family poverty level, which was multiply imputed using six implicates, category-specific n values represent the mean unweighted counts across the six implicates. Survey-weighted percentages, including the distribution of children across poverty categories and the prevalence of SHS exposure within each category, were estimated separately within each implicate and then combined using multiple-imputation procedures. For family poverty level, Rao–Scott chi-square tests were conducted separately within each of the six imputed FPL implicates; *P* < 0.01 in all six implicates.

We estimated survey-weighted mean illness- or injury-missed school days for SHS-exposed and unexposed children for the pooled 2016–2024 sample and by survey year. These descriptive analyses used NSCH survey weights only. Exposure-group differences were estimated using survey-weighted linear regression; year-specific comparisons were obtained from a model including survey year, SHS exposure, and their interaction. Standard errors and tests accounted for NSCH stratification and clustering.

We used propensity score weighting to improve balance in observed characteristics between children with and without SHS exposure (Austin, 2011). For each poverty-level implicate, propensity scores were estimated using logistic regression including child age, sex, race/ethnicity, highest adult education, family poverty level, and survey year. We used average treatment effect on the treated (ATT) weighting, which reweighted unexposed children to resemble exposed children with respect to the above covariates. For pooled analyses, each annual NSCH selected-child survey weight was divided by nine so that the weighted population represented an average annual population during 2016–2024. The rescaled survey weight was then multiplied by the ATT propensity-score weight to obtain the combined analysis weight (Steiner, 2010). Covariate balance was assessed using absolute standardized mean differences (SMD) before and after weighting, with values <0.10 indicating adequate balance. Additional details on propensity-score estimation, weight construction, and diagnostics are provided in the Appendix under “Propensity Score Weighting and ATT Weighting.”

We used a two-part model to estimate the association between SHS exposure and missed school days. The first part used probit regression to model the probability of any missed school days among all children; model-adjusted average marginal effects were obtained from predicted probabilities. The second part used ordinary least squares regression of log-transformed missed school days among children with positive missed days. Predictions from the second part were retransformed to the original scale using Duan's smearing estimator (Duan, 1983). The expected number of missed school days was calculated as the predicted probability of any missed days multiplied by the predicted number of days conditional on having positive missed days. Both parts included SHS exposure and all the covariates, including state and survey-year fixed effects, and were estimated separately for each poverty-level implicate. Implicate-specific estimates were subsequently combined using Rubin's rules (U.S. Census Bureau, 2024).

We used an “excess utilization” approach (Fig. S1 in the Appendix) to estimate excess number of missed school days attributable to SHS exposure (Wang et al., 2018b, Wang et al., 2018a; Yao et al., 2018; Yao et al., 2019). For exposed children, we generated predicted missed school days under the factual condition, i.e., their observed SHS exposure, and under a counterfactual scenario in which SHS exposure was set to zero while all the covariates were retained at their observed values. The difference between these predicted means represents the model-estimated excess missed school days per exposed child.

The relative risks (RRs) of missed school days under the factual and counterfactual conditions estimated from the pooled 2016–2024 model were combined with the survey-weighted 2024 prevalence of SHS exposure to calculate a 2024 prevalence-standardized SHS attributable fraction (SHSAF). Detailed definitions and equations are provided in the Appendix. This calculation assumes that the relative association estimated from the pooled 2016–2024 data is applicable to the 2024 population (Max et al., 2013).

We estimated the total number of missed school days in 2024 in the U.S. by multiplying 2024 public-school enrollment among children aged 6–11 years by the survey-weighted mean number of missed school days per child from the 2024 NSCH. The SHSAF was applied to this total to estimate national SHS-attributable missed school days. We estimated the number of SHS-exposed children by multiplying 2024 public-school enrollment by the survey-weighted prevalence of SHS exposure at home from the 2024 NSCH. The number of attributable days per exposed child was calculated by dividing the national attributable missed school days by the estimated number of exposed children.

Confidence intervals for model-based attributable estimates were obtained using 1000 bootstrap replicates within each poverty-level implicate, with the full propensity-score and outcome-model estimation procedure repeated within each replicate. Bootstrap variances and point estimates were subsequently combined across the six implicates using Rubin's rules. Technical details are provided in the Appendix under “Bootstrap Estimation of Confidence Intervals.”

As an exploratory application, we applied the national model-based RR estimates to California public-school enrollment, SHS prevalence, and attendance-based school-funding information to approximate SHS-attributable missed school days and associated funding loss (California Department of Education, n.d.). Details are provided in the Appendix under “Exploratory California Attendance and School-Funding Application”

We conducted two sensitivity analyses for the open-ended “11 or more days” category: assigning values of 15 and 20 days and fitting a survey-weighted grouped hurdle model using the original categories. To assess whether pandemic-related disruptions to schooling influenced the findings, we also repeated the primary analysis excluding 2020–2021. Propensity scores, ATT weights, and two-part models were re-estimated using data from 2016 to 2019 and 2022–2024, while 2024-specific inputs were retained for the burden estimate. Additional details are provided in the Appendix.

The propensity-score-weighted two-part models used the combined survey–ATT weights (Ridgeway et al., 2015; Wang et al., 2021). The descriptive estimates, 2024 SHS prevalence, and 2024 mean missed school days used the original NSCH survey weights. The two-part models and bootstrap analyses were conducted using Stata version 14.0, and all other analyses were conducted using SAS version 9.4.

This study used publicly available, deidentified data and followed applicable privacy-protection guidelines. The UCSF Institutional Review Board determined that it was not human subjects research; documentation is on file.

## 3 Results

### 3.1 Sample characteristics and unadjusted findings

Table 1 shows that SHS exposure at home among U.S. children aged 6–11 declined over time and averaged 1.63% in 2016–2024. Exposure did not differ significantly by school absence status (1.70% vs 1.48%; *P* = 0.18) but varied across sociodemographic characteristics.

In the pooled 2016–2024 analysis, SHS-exposed children had a higher unadjusted survey-weighted mean number of missed school days than unexposed children (3.50 vs 2.67 days; mean difference, 0.83 days; 95% CI, 0.54, 1.13) (Table 2). Year-specific differences were significant in 2016–2018, 2020, and 2021, ranging from 0.69 to 1.59 days, but not in 2019 or 2022–2024.

**Table 2:** Unadjusted average missed school days among US children aged 6–11 by survey year and secondhand smoke exposure, *n* = 109,030, National Survey of Children's Health (NSCH) 2016–2024.

Survey year	SHS-exposed mean	SE	Unexposed mean	SE	Mean difference	SE	95% CI
2016–2024	3.50	0.15	2.67	0.02	0.83	0.15	(0.54, 1.13)
2016	3.12	0.31	2.44	0.05	0.68	0.32	(0.06, 1.31)
2017	3.85	0.58	2.40	0.07	1.45	0.59	(0.30, 2.59)
2018	3.86	0.56	2.60	0.06	1.26	0.56	(0.15, 2.36)
2019	3.04	0.40	2.68	0.06	0.36	0.41	(−0.43, 1.16)
2020	3.81	0.49	2.23	0.05	1.58	0.49	(0.63, 2.54)
2021	2.87	0.34	1.80	0.04	1.08	0.34	(0.41, 1.74)
2022	3.57	0.32	3.28	0.05	0.29	0.32	(−0.35, 0.92)
2023	4.03	0.53	3.32	0.06	0.71	0.53	(−0.33, 1.75)
2024	3.80	0.45	3.25	0.05	0.55	0.46	(−0.35, 1.44)

Note: SHS indicates secondhand smoke; SE, standard error; and CI, confidence interval. Values are survey-weighted means and standard errors. Mean differences were calculated as SHS-exposed minus unexposed. All estimates used the NSCH selected-child survey weights only; propensity-score weights were not used. The table presents descriptive comparisons without covariate adjustment. The pooled comparison was estimated using survey-weighted linear regression. Year-specific estimates were obtained jointly from a saturated survey-weighted regression including survey year, SHS exposure, and their interaction. Mean differences, standard errors, and 95% CIs were estimated using survey-weighted linear regression comparing exposed and unexposed children.

### 3.2 Adjusted associations and attributable burden

Propensity-score diagnostics indicated good overlap and excellent covariate balance after ATT weighting across all six poverty-level implicates; detailed balance, weight-distribution, and effective-sample-size diagnostics are provided in the Appendix (Tables S1 and Fig. S2).

In adjusted two-part models, SHS exposure was associated with both a higher probability of any missed school days and more missed days among children with positive absences (Table 3). SHS exposure was associated with a 7.00-percentage-point higher probability of any absence (95% CI, 2.00, 11.10 percentage points) and 17.00% more missed days among children with positive absences (ratio, 1.17; 95% CI, 1.10, 1.24).

**Table 3:** Adjusted two-part model results on missed school days among US children aged 6–11, National Survey of Children's Health 2016–2024, *n* = 109,030.

Characteristic/category	Part 1: average marginal effect (95% CI)	Part 2: multiplicative effect (95% CI)
*SHS exposure at home*		
Yes	0.07 (0.02, 0.11)	1.17 (1.10, 1.24)
No	0.00	1.00
*Child sex*		
Male	0.00 (−0.04, 0.04)	1.03 (0.97, 1.09)
Female	0.00	1.00
*Child age*		
Per one-year increase	0.00 (−0.01, 0.01)	0.99 (0.98, 1.01)
*Child race/ethnicity*		
Hispanic	−0.08 (−0.16, 0.00)	0.98 (0.86, 1.12)
Non-Hispanic White	0.00	1.00
Non-Hispanic Black	−0.12 (−0.18, −0.07)	0.95 (0.88, 1.03)
Non-Hispanic Asian	−0.13 (−0.26, −0.01)	0.92 (0.75, 1.14)
Non-Hispanic other/multiracial	−0.01 (−0.07, 0.05)	1.01 (0.92, 1.12)
*Parent's highest education*		
Less than high school	0.00	1.00
High school/vocational/trade/business	0.03 (−0.04, 0.09)	1.01 (0.90, 1.13)
Some college/associate degree	0.05 (−0.02, 0.11)	0.99 (0.89, 1.11)
College degree or above	0.10 (0.02, 0.18)	0.95 (0.83, 1.09)
*Family poverty level*		
<100% FPL	0.00	1.00
100%–199% FPL	0.00 (−0.06, 0.05)	0.99 (0.91, 1.06)
200%–399% FPL	0.03 (−0.03, 0.09)	0.95 (0.87, 1.03)
≥400% FPL	0.01 (−0.09, 0.10)	0.89 (0.78, 1.02)
*Survey year*		
2016	0.00	1.00
2017	−0.03 (−0.11, 0.05)	1.08 (0.94, 1.24)
2018	−0.01 (−0.09, 0.08)	1.13 (1.01, 1.27)
2019	−0.02 (−0.10, 0.07)	1.03 (0.93, 1.14)
2020	−0.06 (−0.13, 0.01)	1.13 (1.01, 1.27)
2021	−0.15 (−0.22, −0.08)	1.05 (0.93, 1.18)
2022	0.01 (−0.05, 0.08)	1.15 (1.03, 1.27)
2023	0.02 (−0.06, 0.10)	1.20 (1.06, 1.36)
2024	0.03 (−0.04, 0.10)	1.19 (1.07, 1.33)

Note: Estimates were obtained separately for each of the six NSCH family-poverty implicates and then combined using Rubin's rules. The combined estimate is the average of the six implicate-specific estimates. Its standard error incorporates both within-implicate sampling variance and between-implicate variation, and the 95% confidence interval was calculated from the resulting total variance. For multiplicative effects, estimates were combined on the logarithmic scale and then exponentiated for presentation. State fixed-effect coefficients are not reported.

Table 4 shows that the predicted mean missed school days among exposed children was 3.50 under observed SHS exposure and 2.72 under the counterfactual no-exposure condition, corresponding to factual and counterfactual RRs of 1.32 and 1.03, respectively. In 2024, SHS prevalence was 1.16%, yielding an SHSAF of 0.34%, meaning that an estimated 0.34% of all missed school days were attributable to SHS exposure at home under the model and stated assumptions. Multiplying 0.34% by the estimated 79.01 million missed school days among 24.24 million U.S. public-school children aged 6–11 years, we calculated that 266,008 days were attributable to SHS exposure, equivalent to 0.95 missed days per exposed child.

**Table 4:** Model-based attributable-burden estimates among the US children aged 6–11, National Survey of Children's Health 2016–2024.

Measure	Estimate	95% CI
Predicted mean missed school days, unexposed children	2.65	(2.57, 2.74)
Predicted mean missed school days, exposed children—factual	3.50	(3.21, 3.79)
Predicted mean missed school days, exposed children—counterfactual no SHS	2.72	(2.60, 2.85)
Excess missed days among exposed children	0.78	(0.46, 1.09)
Factual ratio of predicted mean missed days (factual RR)	1.32	(1.21, 1.44)
Counterfactual ratio of predicted mean missed days (counterfactual RR)	1.03	(0.99, 1.06)
2024 SHS prevalence	1.16%	(0.86%, 1.45%)
Public-school enrollment, ages 6–11	24,237,224	NA
2024 observed mean missed school days	3.26	(3.17, 3.35)
2024 SHS-attributable fraction (SHSAF)	0.34%	(0.18%, 0.50%)
Total national missed school days in 2024	79,012,497	(76,755,922, 81,269,072)
National SHS-attributable missed school days in 2024	266,008	(139,627, 392,390)
National attributable days per exposed child in 2024	0.95	(0.56, 1.34)

Note: The propensity-score and two-part outcome models used combined survey–ATT weights, defined as the product of the NSCH survey weight and the propensity-score-based ATT weight. The 2024 SHS prevalence and observed mean missed school days, which describe the national population rather than the ATT target population, were estimated using the original NSCH survey weights only. The complete estimation procedure was repeated in 1000 bootstrap samples within each of the six family-poverty implicates. Point estimates were calculated separately within each implicate and then averaged. Standard errors and 95% confidence intervals combined the within-implicate bootstrap variance and between-implicate variance using Rubin's rules. Ratios of predicted mean missed school days were combined on the logarithmic scale and exponentiated for presentation. The external 2024 public-school enrollment estimate was treated as fixed; therefore, no confidence interval is reported for enrollment, and uncertainty in enrollment was not incorporated into the confidence intervals for the national burden estimates. CI, confidence interval; ATT, average treatment effect on the treated; RR, ratio of predicted mean missed school days; SHS, secondhand smoke; SHSAF, secondhand-smoke-attributable fraction.

### 3.3 Exploratory and sensitivity analyses

Applying the national model-based RR estimates to California's 2024 SHS prevalence of 0.10% yielded an SHSAF of 0.03% (Table S2). Multiplying 0.03% by the estimated 7.74 million missed school days among 2.49 million public-school children aged 6–11 years, an estimated 2353 missed school days were attributable to SHS exposure, equivalent to 0.91 days per exposed child and approximately $253,759 in attendance-based funding loss in 2024. This exploratory analysis illustrates the potential fiscal implications of SHS-associated absenteeism for schools.

In sensitivity analyses assigning alternative values for the open-ended “11 or more days” category to 15 days and 20 days, burden estimates were 302,844 and 345,573 missed school days; both were within the 95% CI of the primary burden estimate of 266,008 (139,627, 392,390 days) (Appendix Table S3). These findings support the qualitative robustness of the primary conclusion while demonstrating that the absolute burden estimate is moderately sensitive to the numeric value assigned to the open-ended category. In a second sensitivity analysis using the original absence categories, SHS exposure was associated with a 7.00-percentage-point higher probability of any missed days (95% CI, 1.80, 11.10) and 1.63 times the odds of being in a higher absence category among children with positive missed days (95% CI, 1.34, 1.98). SHS exposure was also associated with greater probabilities of higher missed-day categories (Table S3).

After excluding 2020–2021 from the analyses, the association with any absence was no longer statistically significant (average marginal effect, 0.04; 95% CI, −0.01, 0.09), while the multiplicative association among children with any absence remained elevated (1.15; 95% CI, 1.08, 1.24). The corresponding 2024 attributable-burden point estimate was 207,594 missed school days (Table S4).

## 4 Discussion

This study provides the first national estimates of school absenteeism attributable to secondhand smoke (SHS) exposure at home among U.S. children aged 6–11 years. Using nationally representative 2016–2024 NSCH data, we estimated the association between household SHS exposure and school absenteeism and translated it into national SHS-attributable missed school days among children aged 6–11. The positive associations are consistent with earlier regional and national studies (Gilliland et al., 2003; Levy et al., 2011). Recent international evidence also highlights the broader public health relevance of SHS exposure across settings (Hong et al., 2024). Our study extends this literature by quantifying a contemporary national educational burden.

Excluding 2020–2021 attenuated the findings, and the association with any absence became nonsignificant. This may reflect pandemic-related changes, reduced precision after excluding about one-quarter of the overall and SHS-exposed samples, or both. The 2024 attributable-burden point estimate was 207,594 missed school days and remained within the 95% confidence interval of the primary estimate.

Unlike many tobacco-related health outcomes that develop over longer periods, school absenteeism is routinely recorded and may capture shorter-term functional consequences associated with SHS-related illness or symptoms (Allen et al., 2018; Alberg et al., 2003). Because missed school days are also associated with poorer academic performance and increased need for educational support services (Allen et al., 2018), estimating SHS-attributable absenteeism provides a practical measure of the educational impact.

The exploratory California application suggests that SHS-associated absenteeism may also have fiscal implications where school funding is tied to attendance. Although the estimated loss was modest, the findings illustrate potential effects beyond child health. These estimates should be interpreted cautiously because they applied pooled national model estimates to California rather than using a California-specific model.

Our findings support continued efforts to reduce children's SHS exposure at home through smoke-free homes, caregiver cessation support, and broader tobacco control policies (Radó et al., 2021; Rosen et al., 2014). School absenteeism may also provide an additional population-level indicator of these benefits.

Several limitations should be considered. First, SHS exposure and illness- or injury-related school absences were parent-reported and may be subject to recall, social-desirability, and correlated reporting errors. The direction of bias is uncertain because exposure misclassification may affect exposure prevalence, the exposure–outcome association, and the attributable fraction differently. Second, missed school days were reported categorically. Assigning category midpoints and 11 days to the open-ended ≥11-day category may not fully represent the underlying distribution of absenteeism. However, sensitivity analyses using alternative values and a grouped model retaining the original categories produced similar findings. Third, residual confounding from unmeasured household-level or time-varying state-specific factors cannot be excluded despite propensity-score weighting and covariate adjustment. Fourth, the pooled 2016–2024 association was applied to the 2024 population because annual exposed samples were limited, assuming that the pooled association was applicable to 2024; estimates for NSCH school-enrolled children were also extrapolated using public-school enrollment, creating some mismatch between the modeled and extrapolated populations. Fifth, pandemic-related changes in schooling and attendance may have influenced the estimated association. After excluding 2020–2021, the association with any absence became nonsignificant and the overall and SHS-exposed samples were reduced by about 25%. So the attenuation may reflect pandemic-related changes, reduced precision, or both. Finally, the cross-sectional design limits temporal and causal interpretation because the timing and duration of SHS exposure relative to absences cannot be established. The attributable estimates also assume sufficient overlap between exposed and comparable unexposed children and correct model specification and should therefore be interpreted as model-based estimates under these assumptions.

## 5 Conclusions

In conclusion, SHS exposure was associated with increased school absenteeism among U.S. children and an estimated 266,008 SHS-attributable missed school days in 2024. Reducing children's SHS exposure at home may improve school attendance while supporting both child health and the efficient use of educational resources.

## CRediT authorship contribution statement

**Yingning Wang:** Writing – review & editing, Writing – original draft, Validation, Supervision, Project administration, Methodology, Investigation, Funding acquisition, Formal analysis, Data curation, Conceptualization. **Hai-Yen Sung:** Writing – review & editing, Methodology, Conceptualization. **Wendy Max:** Writing – review & editing, Methodology, Conceptualization. **Bonnie Halpern-Felsher:** Writing – review & editing, Conceptualization. **Dian Gu:** Writing – review & editing, Methodology. **Sarah Planche:** Writing – review & editing, Conceptualization. **Chunxia Wang:** Writing – review & editing, Methodology.

## Funding

This work was supported by the Tobacco-Related Disease Research Program (TRDRP), University of California Office of the President [Grant No. T34IR8235]. The content is authors' responsibility and does not necessarily represent the views of TRDRP or the University of California. The authors declare no conflicts of interest.

## Declaration of competing interest

The authors declare that they have no known competing financial interests or personal relationships that could have appeared to influence the work reported in this paper.

## Data Availability

Data are publicly available from the U.S. Census Bureau's National Survey of Children's Health (NSCH): https://www.census.gov/programs-surveys/nsch/data/

## Appendix A Supplementary data

Supplementary material: Additional methods, tables, figures, and sensitivity analyses.
