# Supplementary material for: Secondhand smoke exposure at home and school absenteeism among children in the United States: national estimates of attributable missed school days
Source: Prev Med Rep. 2026 Sep 13;71:103636. doi: 10.1016/j.pmedr.2026.103636 (PMC13602684; doi:10.1016/j.pmedr.2026.103636)
Supplement: Supplementary file 1 [file mmc1.docx]

**Secondhand Smoke Exposure at Home and School Absenteeism Among Children in the United States: National Estimates of Attributable Missed School Days**

**Appendix**

1. **NSCH Sampling, Survey Weights, and Multiple Imputation**

The NSCH uses an address-based, stratified sampling design conducted separately within each state and the District of Columbia. Residential addresses are stratified using administrative information indicating the likelihood that children reside in the household, with different address-selection probabilities across strata. A household screener identifies resident children aged 0–17 years and collects basic demographic and health information. If multiple eligible children reside in the household, one child is subsampled for the age-specific topical questionnaire. The within-household selection procedure oversamples children with special health care needs and, secondarily, children aged 0–5 years; thus, an individual child’s selection probability depends on the number and characteristics of eligible children in the household. Child sex is not used in within-household child selection but is incorporated, along with other demographic characteristics, in calibration of the final survey weights.

The NSCH selected-child topical survey weight incorporates the probabilities of address selection and within-household child selection, adjustments for screener and topical-questionnaire nonresponse, and calibration to external population benchmarks. Therefore, no additional adjustment for within-household child selection was required. For pooled 2016–2024 analyses, each annual selected-child survey weight was divided by nine. This rescaling preserves relative weights while making the pooled weighted population represent an average annual population rather than the sum of nine annual populations.

Family poverty ratio is supplied in the NSCH public-use files as six multiply imputed continuous values, or implicates. Missing family income used to construct family poverty ratio is imputed by the NSCH using sequential regression methods. For each implicate, we categorized family poverty ratio as <100%, 100%–199%, 200%–399%, or ≥400% of the federal poverty level (FPL).

All analyses involving family poverty level were conducted separately within each of the six implicates. Implicate-specific estimates and variances were then combined using Rubin’s rules, following NSCH guidance. For descriptive Table 1 counts, category-specific unweighted sample sizes were calculated within each implicate and reported as the mean unweighted counts across the six implicates. Survey-weighted percentages were estimated separately within each implicate and combined using multiple-imputation procedures. Rao–Scott tests of the association between poverty level and SHS exposure were conducted within each implicate.

2. **Additional Descriptive Analyses**

Table 1 descriptive estimates were calculated using NSCH selected-child survey weights only. Rao–Scott chi-square tests were used to evaluate bivariate associations between household SHS exposure and categorical characteristics. For family poverty level, percentages and their variances were estimated separately within each of the six implicates and combined using multiple-imputation procedures. Because children with imputed family poverty ratio could fall into different categories across implicates, there is no single exact category-specific unweighted count; Table 1 therefore reports the mean unweighted count across the six implicates.

For Table 2, survey-weighted mean missed school days were estimated for SHS-exposed and unexposed children without propensity-score weighting or additional covariate adjustment. “Unadjusted” therefore refers to the absence of propensity-score and regression covariate adjustment, not to an absence of survey weighting. Pooled comparisons were estimated using survey-weighted linear regression. Year-specific means and exposed-minus-unexposed comparisons were obtained jointly from a saturated model containing survey year, SHS exposure, and their interaction. Standard errors accounted for survey stratification and household clustering, and P values were obtained from design-adjusted Wald tests of the corresponding regression contrasts.

3. **Propensity Score Weighting and ATT Weighting**

Propensity-score models were estimated separately for each family poverty-level implicate using weighted logistic regression, with household SHS exposure as the dependent variable. Covariates included child age, sex, race/ethnicity, highest education level among reported adults, family poverty level, and survey year. The models were estimated using the rescaled average-annual NSCH selected-child survey weights. Individual state indicators were not included in the propensity-score model because the low prevalence of household SHS exposure resulted in complete separation in some state-specific bootstrap samples. State and survey-year fixed effects were retained in both components of the two-part outcome model.

ATT propensity weights were constructed from the predicted propensity score, e(X). Exposed children received an ATT weight of 1. Unexposed children received a weight of e(X)/[1−e(X)], thereby reweighting the unexposed group to resemble the exposed group in measured characteristics. The final combined analysis weight was calculated as:

Combined analysis weight = rescaled NSCH selected-child survey weight × ATT propensity weight.

No additional stabilization, normalization, or truncation of the ATT propensity weights was applied in the primary analysis.

Propensity-score diagnostics were evaluated separately within each poverty-level implicate. We assessed propensity-score overlap, ATT weight distributions, absolute standardized mean differences before and after weighting, and effective sample size. Absolute standardized mean differences <0.10 were considered evidence of adequate covariate balance. Effective sample size (ESS) for the combined analysis weights was calculated as: ESS = (Σwᵢ)² / Σwᵢ², where wᵢ denotes the combined analysis weight. Diagnostic results were summarized as ranges or maximum values across the six implicates and are presented in Appendix Figure S2 and Table S1.

4. **Two-Part Outcome Model and Prediction**

The first component of the two-part model used probit regression to estimate the probability of having any illness- or injury-related missed school days. Adjusted probabilities were obtained by post-estimation prediction, and average marginal effects were calculated for the SHS exposure variable.

The second component was estimated among children with positive missed school days using ordinary least squares regression of the natural logarithm of missed school days. The logarithmic transformation was used because positive missed days were right-skewed. Coefficients were exponentiated to obtain multiplicative effects on positive missed school days. Predicted values were retransformed to the original day scale using Duan’s smearing estimator.

For each child, the expected number of missed school days was calculated as the product of the predicted probability of any missed days from the first component and the retransformed predicted number of days conditional on having positive missed days from the second component.

Both components included household SHS exposure, child age, sex, race/ethnicity, highest adult education, family poverty level, state fixed effects, and survey-year fixed effects. Models were estimated separately within each poverty-level implicate. First-part estimates and variances were combined across implicates using Rubin’s rules. Second-part coefficients were combined on the fitted log scale and subsequently exponentiated.

5. **Factual and Counterfactual Predicted Means**

For each poverty-level implicate, model-based predictions were generated under factual and counterfactual exposure scenarios. Under the factual scenario, predictions were calculated using each child’s observed household SHS exposure and observed covariate values.

For the counterfactual scenario, household SHS exposure was set to zero for exposed children while all other observed covariate values were retained. Predictions for unexposed children remained unchanged. Among exposed children, we calculated the mean predicted number of missed school days under observed exposure and the counterfactual mean predicted after setting exposure to zero. The difference between these means represents model-estimated excess missed school days per exposed child in the ATT-standardized population.

For the ratio-based attributable-fraction calculation, the factual relative risk was defined as the mean predicted missed school days among exposed children under observed SHS exposure divided by the mean predicted missed school days among unexposed children. The counterfactual relative risk was defined using predictions for the same exposed children after setting SHS exposure to zero, divided by the same unexposed-group predicted mean. In this analysis, these RRs refer to ratios of predicted mean missed school days rather than risk ratios for a binary outcome.

The pooled 2016–2024 factual and counterfactual ratios were combined with the survey-weighted prevalence of household SHS exposure in the 2024 NSCH to calculate the 2024 prevalence-standardized SHS-attributable fraction (SHSAF), as specified in Equation A1. This approach uses the more precise relative contrast estimated from all nine survey years while allowing the prevalence component to reflect the 2024 population. It assumes that the pooled 2016–2024 SHS–absenteeism association is applicable to the 2024 population.

**Figure S1: Framework of deriving secondhand smoke-attributable missed school days**

**SHS-exposure-attributable fraction (AF)**

$$\boldsymbol{=}\frac{\boldsymbol{US prevalence*(}\boldsymbol{RR}_{\boldsymbol{0}}\boldsymbol{-}\boldsymbol{RR}_{\boldsymbol{1}}\boldsymbol{)}}{\boldsymbol{Total factual predictions}}$$

**Factual predicted missed school days**

**Relative risks (RR) for factual case,** $\boldsymbol{RR}_{\boldsymbol{0}}$

**Counterfactual predicted missed school days**

**RR for counterfactual case,** $\boldsymbol{RR}_{\boldsymbol{1}}$

**US SHS home exposure estimates**

**Use NSCH survey to estimate the relationship between SHS home exposure and absenteeism among children (aged 6-11) in the US**

**Total annual missed school days for children aged 6-11**

**US annual SHS-exposure-attributable missed school days**

6. **SHS-Attributable Fraction**

$SHSAF=\frac{P_{SHS}*u_{SHS}- u_{SHS\to n})}{{(1-P}_{SHS}){*u}_{0} {+ P}_{SHS}*u_{SHS}}$(Equation A1)

$SHSAF=\frac{P_{SHS}*({RR}_{SHS}- {RR}_{SHS\to n})}{P_{SHS}{*RR}_{SHS} {+ (1-P}_{SHS} )}$ (Equation A2)

Where

$P_{SHS},$= prevalence of SHS exposure.

$u_{SHS}$= the predicted mean among exposed children under their factual exposure

$u_{SHS\to n}$= the predicted mean among exposed children under their counterfactual exposure

$u_{0}$ =	the predicted mean for the reference group—unexposed group

${RR}_{SHS}$	= relative risk of missed school days for factual condition, defined by the ratio of the mean predicted missed school days for children with SHS exposure to the mean predicted missed school days for children without SHS exposure.

${RR}_{SHS\to n}$	=relative risk of missed school days for counterfactual condition, defined by the ratio of the counterfactual predicted mean among exposed children after setting household SHS exposure to zero to the predicted mean among unexposed children.

Equation A1 can be expressed equivalently using ratios of predicted missed school days. Dividing the numerator and denominator by $u_{0}$ gives Equation A2. Equations A1 and A2 are algebraically equivalent. Equation A1 presents the SHSAF directly as excess predicted missed days divided by total factual predicted missed days, whereas Equation A2 expresses the same quantity using ratios of predicted means.

7. **Estimation of National SHS-Attributable Missed School Days**

Let $N$ denote the estimated number of public-school children aged 6–11 years in 2024, obtained from the National Center for Education Statistics; $\bar{Y}$denote the survey-weighted mean number of missed school days per child, estimated using the 2024 NSCH; $P_{e}$ denote the survey-weighted prevalence of SHS exposure; and $SHSAF$ denote the SHS-attributable fraction. The national total number of missed school days was estimated as:

$$T=N\times\bar{Y}$$

The national number of SHS-attributable missed school days was estimated as:

$$T_{\left\{ SHS \right\}}=SHSAF\times T=SHSAF\times N\times\bar{Y}$$

The number of SHS-exposed children was estimated as:

$$N_{e}=N\times P_{e}$$

Finally, the average number of SHS-attributable missed school days per exposed child was estimated as:

$$\bar{Y}_{SHS,e}=\frac{T_{SHS}}{N_{e}}=\frac{SHSAF\times N\times\bar{Y}}{N\times P_{e}}$$

8. **Bootstrap Estimation of Confidence Intervals**

We used a full-pipeline, stratified household-PSU bootstrap with 1,000 replicates per poverty-level implicate to estimate uncertainty for the factual and counterfactual predicted means, excess predicted missed school days, ratios of predicted means, the 2024 SHS-attributable fraction (SHSAF), national attributable missed school days, and attributable days per exposed child. Within each replicate, we re-estimated the propensity-score model, reconstructed the ATT and combined analysis weights, refitted both components of the two-part model, recalculated the Duan smearing factor, and regenerated all factual, counterfactual, and burden estimates.

The 2024 SHSAF was recalculated within each replicate by combining the factual and counterfactual ratios of predicted means from the pooled 2016–2024 model with the survey-weighted 2024 prevalence of household SHS exposure estimated in that replicate. The survey-weighted mean number of missed school days and the number of exposed children in 2024 were also recalculated within each replicate. The 2024 public-school enrollment estimate, obtained from an external source, was treated as fixed; therefore, the bootstrap captured sampling and model-estimation uncertainty from the NSCH but not uncertainty in the external enrollment estimate.

Household PSUs were sampled with replacement within survey strata. Because restriction to the final analytic sample resulted in a small number of strata containing only one PSU, affected strata were pooled within state-year cells for bootstrap resampling only. The propensity-score and outcome analyses otherwise retained the original NSCH survey-design information, including the original design strata and survey weights, with bootstrap-generated PSU identifiers used to identify resampled clusters. The single-unit centering adjustment was retained.

The same sequence of PSU resamples was used across the six poverty-level implicates to preserve alignment of bootstrap samples. For each estimand, the bootstrap variance within each implicate was treated as the within-imputation variance. The six original-sample point estimates, within-imputation bootstrap variances, and between-imputation variance were then combined using Rubin’s rules. Ratios of predicted means were combined on the logarithmic scale and exponentiated after combination. Original full-sample estimates were reported as point estimates, while the bootstrap distributions were used to estimate within-imputation uncertainty.

We monitored every replicate for estimation failure, including propensity-score or outcome-model nonconvergence, absence of an exposure or outcome category, invalid predictions, and extreme or undefined weights. Replicates with an estimation error or an undefined target estimate were recorded as failed and were not used to calculate the within-imputation variance. All 1,000 requested replicates were completed successfully in each of the six poverty implicates, yielding 6,000 successful replicate analyses and no failed or excluded replicates. Thus, the success rate was 100% for every implicate. No replicate was excluded because of a large ATT weight, and no replicate replacement was required.

9. **Exploratory California Attendance and School-Funding Application**

For the exploratory California application, we retained the factual and counterfactual relative contrasts estimated from the pooled national 2016–2024 model and combined them with the 2024 California household SHS prevalence to calculate a California prevalence-standardized SHSAF. California public-school enrollment and California-specific 2024 survey-weighted mean missed school days were then used to approximate SHS-attributable missed school days among California children aged 6–11 years.

The corresponding attendance-based funding loss was estimated by multiplying attributable missed school days by the applicable California educational funding amount per average daily attendance day obtained from the California Department of Education. This calculation was intended only to illustrate potential fiscal implications and did not represent a separate California-specific regression analysis.

10. **Sensitivity Analyses**

We conducted two sensitivity analyses to assess the influence of the open-ended “11 or more days” response category for missed school days. First, we repeated the primary analyses assigning alternative values of 15 and 20 days to this category, instead of the value used in the primary analysis, and re-estimated the predicted means and SHS-attributable burden. Second, we fitted a survey-weighted grouped hurdle model that retained the original absence categories (1–3, 4–6, 7–10, and ≥11 days) without assigning exact day values. The first component modeled the probability of any missed school days, and the second used an ordinal logistic model among children with positive missed days to estimate the odds of being in a higher absence category. These sensitivity analyses used the same weighting, covariate adjustment, and multiple-imputation procedures as the primary analyses.

In addition to these outcome-coding sensitivity analyses, we conducted a separate sensitivity analysis to assess the potential influence of the COVID-19 pandemic period. The propensity-score model and ATT weights were re-estimated separately within each family poverty-level implicate after excluding 2020–2021. The two-part outcome models, Duan smearing retransformation, and factual and counterfactual predictions were then re-estimated using data from 2016–2019 and 2022–2024. The 2024 attributable-burden calculation continued to use the 2024-specific SHS prevalence, mean missed school days, and public-school enrollment.

**Figure S2. Propensity-score distributions among children exposed and unexposed to household secondhand smoke across the six multiply imputed family poverty level implicates.** The propensity-score model was estimated separately within each implicate using the National Survey of Children’s Health selected-child survey weights. The panels show similar distributions across implicates and substantial overlaps between exposure groups, although unexposed children were more concentrated at low propensity scores. FPL, federal poverty level; SHS, secondhand smoke.

| **Table S1. Propensity-score balance, average treatment effect on the treated-weight distributions, and effective sample size across six family poverty-level implicates** | | | | | | | |
| --- | --- | --- | --- | --- | --- | --- | --- |
| **Panel A Covariate balance before and after ATT weighting across six FPL implicates** | | | | | | | |
| Covariate | | SMD before, range | Maximum \|SMD\| before | SMD after, range | Maximum \|SMD\| after | All <0.10 | |
| Male | | 0.052 to 0.052 | 0.052 | 0.001 to 0.001 | 0.001 | Yes | |
| Age, years | | 0.193 to 0.193 | 0.193 | −0.002 to −0.001 | 0.002 | Yes | |
| Race and ethnicity: Hispanic | | −0.384 to −0.384 | 0.384 | −0.001 to −0.001 | 0.001 | Yes | |
| Race and ethnicity: non-Hispanic White | | 0.033 to 0.033 | 0.033 | 0.004 to 0.005 | 0.005 | Yes | |
| Race and ethnicity: non-Hispanic Black | | 0.383 to 0.383 | 0.383 | −0.003 to −0.003 | 0.003 | Yes | |
| Race and ethnicity: non-Hispanic Asian | | −0.221 to −0.221 | 0.221 | −0.001 to 0.000 | 0.000 | Yes | |
| Race and ethnicity: other/multiracial | | 0.076 to 0.076 | 0.076 | −0.003 to −0.002 | 0.003 | Yes | |
| Adult education: less than high school | | 0.171 to 0.171 | 0.171 | 0.005 to 0.006 | 0.006 | Yes | |
| Adult education: high school/vocational | | 0.548 to 0.548 | 0.548 | −0.002 to −0.002 | 0.002 | Yes | |
| Adult education: some college/associate degree | | 0.249 to 0.249 | 0.249 | −0.002 to −0.002 | 0.002 | Yes | |
| Adult education: college degree or higher | | −0.968 to −0.968 | 0.968 | 0.001 to 0.001 | 0.001 | Yes | |
| Family poverty level: <100% FPL | | 0.551 to 0.607 | 0.607 | −0.001 to 0.000 | 0.001 | Yes | |
| Family poverty level: 100%–199% FPL | | 0.276 to 0.320 | 0.320 | 0.000 to 0.001 | 0.001 | Yes | |
| Family poverty level: 200%–399% FPL | | −0.298 to −0.250 | 0.298 | 0.000 to 0.000 | 0.000 | Yes | |
| Family poverty level: ≥400% FPL | | −0.742 to −0.726 | 0.742 | 0.000 to 0.001 | 0.001 | Yes | |
| Survey year: 2016 | | 0.191 to 0.191 | 0.191 | 0.000 to 0.001 | 0.001 | Yes | |
| Survey year: 2017 | | −0.071 to −0.071 | 0.071 | 0.001 to 0.002 | 0.002 | Yes | |
| Survey year: 2018 | | 0.062 to 0.062 | 0.062 | 0.002 to 0.002 | 0.002 | Yes | |
| Survey year: 2019 | | 0.001 to 0.001 | 0.001 | 0.001 to 0.001 | 0.001 | Yes | |
| Survey year: 2020 | | 0.028 to 0.028 | 0.028 | −0.003 to −0.002 | 0.003 | Yes | |
| Survey year: 2021 | | 0.004 to 0.004 | 0.004 | −0.001 to 0.000 | 0.000 | Yes | |
| Survey year: 2022 | | −0.039 to −0.039 | 0.039 | −0.002 to −0.001 | 0.002 | Yes | |
| Survey year: 2023 | | −0.106 to −0.106 | 0.106 | 0.000 to 0.000 | 0.000 | Yes | |
| Survey year: 2024 | | −0.112 to −0.112 | 0.112 | 0.000 to 0.000 | 0.000 | Yes | |
| Note: SMD, standardized mean difference. Positive values indicate a higher covariate mean or proportion among exposed children; negative values indicate a lower value. Balance was evaluated separately in each of the six FPL implicates. Absolute SMD <0.10 was considered acceptable. State indicators were not part of the final propensity-score model and are not included in this table. | | | | | | | |
| **Panel B. ATT-weight distributions and effective sample sizes by FPL implicate** | | | | | | | |
| Family poverty level | Mean ATT weight, unexposed | P95 ATT weight, unexposed | P99 ATT weight, unexposed | Maximum ATT weight, unexposed | ESS exposed | ESS unexposed | ESS overall |
| 1 | 0.01380 | 0.06264 | 0.09821 | 0.17484 | 516 | 7,213 | 1,926 |
| 2 | 0.01382 | 0.06255 | 0.09715 | 0.17379 | 516 | 7,330 | 1,928 |
| 3 | 0.01384 | 0.06277 | 0.09529 | 0.16904 | 516 | 7,384 | 1,929 |
| 4 | 0.01387 | 0.06261 | 0.09809 | 0.17601 | 516 | 7,518 | 1,931 |
| 5 | 0.01387 | 0.06168 | 0.09676 | 0.17414 | 516 | 7,489 | 1,931 |
| 6 | 0.01382 | 0.06171 | 0.09614 | 0.17147 | 516 | 7,379 | 1,929 |
| Note: ATT, average treatment effect on the treated; ESS, effective sample size; FPL, federal poverty level. Exposed children received an ATT weight of 1. Unexposed children received e(X)/[1−e(X)]. ESS values were calculated from the final combined NSCH survey × ATT weights. | | | | | | | |

| **Table S2. Exploratory California estimates of secondhand smoke exposure-attributable missed school days and attendance-based funding loss, 2024** | |
| --- | --- |
| Measure | Estimate |
| Predicted mean missed days among exposed children under observed exposure | 3.50 days |
| Predicted mean missed days among exposed children under counterfactual no exposure | 2.72 days |
| Predicted mean missed days among unexposed children | 2.65 days |
| Factual ratio of predicted mean missed days | 1.32 |
| Counterfactual ratio of predicted mean missed days | 1.03 |
| California household SHS prevalence, 2024 | 0.10% |
| California SHS-attributable fraction | 0.03% |
| California public-school children aged 6–11 years, 2024 | 2,491,111 |
| Mean missed school days per child in California, 2024 | 3.11 days |
| Estimated total missed school days in California, 2024 | 7,737,022 days |
| Estimated SHS-exposed children in California, 2024 | 2,593 |
| SHS-attributable missed school days | 2,353 days |
| SHS-attributable days per exposed child | 0.91 days |
| Attendance-based funding amount per missed day | $107.86 |
| Estimated attendance-based funding loss | $253,759 |
| Note: SHS, secondhand smoke. The factual and counterfactual ratios were estimated from the national 2016–2024 NSCH model and applied to the 2024 California household SHS prevalence. The California SHS-attributable fraction was calculated as p*(RR factual − RR counterfactual)/[(1 − p) + p*(RR factual)]. Total missed school days equaled California public-school enrollment multiplied by the estimated 2024 mean missed days. SHS-attributable days equaled total missed days multiplied by the California SHS-attributable fraction. Funding loss was calculated using the unrounded attributable-day estimate. This exploratory application was not based on a separate California-specific regression analysis. Calculations used unrounded prevalence, attributable-fraction, and model estimates; displayed values are rounded. | |

| **Table S3: Sensitivity Analyses of Household Secondhand Smoke Exposure and Missed School Days Using Alternative Coding of the Open-Ended Absence Category** | | | | | | |
| --- | --- | --- | --- | --- | --- | --- |
| **I: Sensitivity analyses using alternative values for the “11 or more days” missed-school category** | | | | | | |
| **Panel A: Two-part-model estimates under alternative values for the “11 or more days” category** | | | | | | |
| Value assigned to “11 or more days” | Part 1: average marginal effect (95% CI) | Part 2: multiplicative effect (95% CI) | | | | |
| 11 days (primary) | 0.07 (0.02, 0.11) | 1.17 (1.10, 1.24) | | | | |
| 15 days | 0.07 (0.02, 0.11) | 1.19 (1.11, 1.27) | | | | |
| 20 days | 0.07 (0.02, 0.11) | 1.21 (1.12, 1.30) | | | | |
| Note: SHS, secondhand smoke; TPM, two-part model. Part 1 models the probability of any missed school days. Because assigning 11, 15, or 20 days to the open-ended positive category does not change the binary indicator of any missed days, the Part 1 coefficient and average marginal effect are identical across specifications and are carried forward from the primary model. Part 2 models the log number of missed days conditional on having any missed days. Estimates were combined across six family-poverty implicates using Rubin’s rules. | | | | | | |
| **Panel B: Predicted means and 2024 SHS-attributable burden under alternative values for the “11 or more days” category** | | | | | | |
| Measure | 11 days (primary) | 15 days | | 20 days | | |
| Predicted mean among unexposed children, days | 2.65 | 2.86 | | 3.12 | | |
| Predicted mean among exposed children—factual, days | 3.50 | 3.83 | | 4.24 | | |
| Predicted mean among exposed children—counterfactual no SHS, days | 2.72 | 2.94 | | 3.20 | | |
| Excess predicted days among exposed children | 0.78 | 0.90 | | 1.04 | | |
| Factual ratio of predicted mean missed days | 1.32 | 1.34 | | 1.36 | | |
| Counterfactual ratio of predicted mean missed days | 1.03 | 1.03 | | 1.03 | | |
| 2024 household SHS prevalence | 1.16% | 1.16% | | 1.16% | | |
| 2024 observed mean missed school days | 3.26 | 3.46 | | 3.72 | | |
| 2024 SHS-attributable fraction | 0.34% | 0.36% | | 0.38% | | |
| Total national missed school days in 2024 | 79,012,497 | 83,943,801 | | 90,107,930 | | |
| National SHS-attributable missed school days | 266,008 | 302,844 | | 345,573 | | |
| National attributable days per exposed child | 0.95 | 1.08 | | 1.23 | | |
| Note: Burden values are combined point estimates across six poverty implicates. The 2024 SHS-attributable fraction uses the 2024 survey-weighted SHS prevalence and the pooled 2016–2024 model-estimated factual and counterfactual ratios. | | | | | | |
| **II: Grouped hurdle sensitivity analysis of household SHS exposure and missed school days** | | | | | | |
| **Panel A. Grouped hurdle model estimates** | | | | | | |
| Characteristic / category | Part 1: average marginal effect (95% CI) | | | Part 2: proportional odds ratio (95% CI) | | |
| SHS exposure at home |  | | |  | | |
| Yes | 0.07 (0.02, 0.11) | | | 1.63 (1.34, 1.98) | | |
| No | Reference | | | Reference | | |
| **Panel B. Adjusted predicted probabilities of positive missed-day categories** | | | | | | |
| Positive missed-day category | No SHS: probability (95% CI) | SHS: probability (95% CI) | | Adjusted probability difference (95% CI) | | |
| 1–3 days | 0.57 (0.56, 0.59) | 0.46 (0.41, 0.50) | | −0.12 (−0.16, −0.07) | | |
| 4–6 days | 0.25 (0.22, 0.27) | 0.28 (0.25, 0.31) | | 0.04 (0.02, 0.05) | | |
| 7–10 days | 0.10 (0.09, 0.12) | 0.14 (0.12, 0.16) | | 0.04 (0.02, 0.05) | | |
| ≥11 days | 0.08 (0.07, 0.09) | 0.12 (0.10, 0.15) | | 0.04 (0.02, 0.06) | | |
| Note: Estimates were obtained separately for each of the six NSCH family poverty level implicates and then combined using Rubin’s rules. SHS, secondhand smoke. Part 1 estimates the average marginal effect of household SHS exposure on the probability of any missed school days. Part 2 is a proportional-odds model fitted among children with at least one missed school day; the odds ratio compares the odds of being in a higher missed-day category for SHS-exposed versus unexposed children. Panel B probabilities are adjusted predictions conditional on having any missed school days. Probability differences were calculated as exposed minus unexposed. Estimates and standard errors were combined across six family poverty level implicates using Rubin’s rules. Unlike the primary log-linear TPM, the grouped model does not assign exact numeric values to the positive categories and therefore does not directly estimate expected or attributable numbers of missed school days. | | | | | | |
| **Table S4. Sensitivity Analysis Excluding the 2020–2021 Survey Years, National Survey of Children’s Health, 2016–2019 and 2022–2024** | | | | | |  |
| **Measure** | | | **Primary analysis, 2016–2024** | | **Excluding 2020–2021** |  |
| Part 1: SHS average marginal effect on any absence | | | 0.07 (0.02, 0.11) | | 0.04 (−0.01, 0.09) |  |
| Part 2: multiplicative effects among children with any absence | | | 1.17 (1.10, 1.24) | | 1.15 (1.08, 1.24) |  |
| Predicted mean missed days, unexposed children | | | 2.65 | | 2.82 |  |
| Predicted mean missed days, exposed children, factual | | | 3.50 | | 3.55 |  |
| Predicted mean missed days, exposed children, counterfactual no SHS | | | 2.72 | | 2.90 |  |
| Excess missed days among exposed children | | | 0.78 | | 0.64 |  |
| Factual ratio of predicted mean missed days | | | 1.32 | | 1.26 |  |
| Counterfactual ratio of predicted mean missed days | | | 1.03 | | 1.03 |  |
| 2024 SHS-attributable fraction, % | | | 0.34 | | 0.26 |  |
| 2024 SHS-attributable missed school days | | | 266,008 | | 207,594 |  |
| Attributable missed days per exposed child | | | 0.95 | | 0.74 |  |
| Note: SHS, secondhand smoke. For Part 1 and Part 2, 95% confidence intervals are presented in parentheses after the point estimates. Part 1 reports the average marginal effect of SHS exposure on the probability of any missed school days. Part 2 reports the exponentiated coefficient from the log-linear model among children with any missed school days. Estimates and variances were combined across the six family poverty-level implicates using Rubin’s rules. All other values are combined point estimates across the six implicates. The 2024 SHS-attributable fraction uses the 2024 survey-weighted SHS prevalence. | | | | | |  |
